# Cost-effectiveness analysis of case management for optimized antithrombotic treatment in German general practices compared to usual care – results from the PICANT trial

**DOI:** 10.1186/s13561-019-0221-2

**Published:** 2019-02-07

**Authors:** Lisa R. Ulrich, Juliana J. Petersen, Karola Mergenthal, Andrea Berghold, Gudrun Pregartner, Rolf Holle, Andrea Siebenhofer

**Affiliations:** 1 Institute of General Practice, Goethe-University Frankfurt am Main, Frankfurt, Germany; 2 Institute for Medical Informatics, Statistics and Documentation, Medical University of Graz, Graz, Austria; 3 Helmholtz Zentrum München - German Research Center for Environmental Health, Institute of Health Economics and Health Care Management, Neuherberg, Germany; 4 German Center for Diabetes Research, Neuherberg, Germany; 5 Institute of General Practice and Evidence-Based Health Services Research, Medical University of Graz, Graz, Austria

**Keywords:** Anticoagulants [MeSH], Chronic disease [MeSH], Cost-effectiveness analysis, Primary health care [MeSH], Case management [MeSH], Health services research [MeSH]

## Abstract

**Background:**

By performing case management, general practitioners and health care assistants can provide additional benefits to their chronically ill patients. However, the economic effects of such case management interventions often remain unclear although how to manage the burden of chronic disease is a key question for policy-makers. This analysis aimed to compare the cost-effectiveness of 24 months of primary care case management for patients with a long-term indication for oral anticoagulation therapy with usual care.

**Methods:**

This analysis is part of the cluster-randomized controlled Primary Care Management for Optimized Antithrombotic Treatment (PICANT) trial. A sample of 680 patients with German statutory health insurance was initially considered for the cost analysis (92% of all participants at baseline). Costs included all disease-related direct health care costs from the payer’s perspective (German statutory health insurers) plus case management costs for the intervention group. A-Quality Adjusted Life Year (QALY) measurement (EQ-5D-3 L instrument) was used to evaluate utility, and incremental cost-effectiveness ratio (ICER) to assess cost-effectiveness. Mean differences were calculated and displayed with 95%-confidence intervals (CI) from non-parametric bootstrapping (1000 replicates).

**Results:**

*N* = 505 patients (505/680, 74%) were included in the cost analysis (complete case analysis with a follow-up after 12 and 24 months as well as information on cost and QALY). After two years, the mean difference of direct health care costs per patient (€115, 95% CI [− 201; 406]) and QALYs (0.03, 95% CI [− 0.04; 0.11]) in the two groups was small and not significant. The costs of case management in the intervention group caused mean total costs per patient in this group to rise significantly (mean difference €503, 95% CI [188; 794]). The ICER was €16,767 per QALY. Regardless of the willingness of insurers to pay per QALY, the probability of the intervention being cost-effective never rose above 70%.

**Conclusions:**

A primary care case management for patients with a long-term indication for oral anticoagulation therapy improved QALYs compared to usual care, but was more costly. However, the results may help professionals and policy-makers allocate scarce health care resources in such a way that the overall quality of care is improved at moderate costs, particularly for chronically ill patients.

**Trial registration:**

Current Controlled Trials ISRCTN41847489.

## Background

In Germany, general practitioners (GPs) are responsible for managing lifelong oral anticoagulation (OAC) therapy for the majority of patients [1]. Most such patients suffer from chronic conditions such as atrial fibrillation / flutter, recurrent venous and / or pulmonary thromboembolisms, or have mechanical heart prostheses [2]. They are generally treated with coumarins, or the direct oral anticoagulants (DOACs) dabigatran, rivaroxaban, apixaban, and edoxaban that have been shown to be effective in preventing thromboembolic complications [3] and reducing the risk of stroke [4]. Care for patients with (multiple) chronic conditions is quickly becoming a dominant health and economic burden for almost all health care systems [5] and effective interventions are necessary to meet their needs [6]. Patients with complex and chronic conditions can benefit considerably from the provision of care by team-based and inter-professional collaborative health care management [7, 8], in which different health care professions such as medical doctors, health care assistants (HCAs), nurse practitioners, and physician assistants cooperate [9] at modest incremental costs [10]. In Germany, general practices generally employ one or more HCAs. They receive 2 years of basic vocational training and usually perform administrative tasks and deliver basic medical care. Even though health care assistants do not have similar academic qualifications to physician assistants and nurse practitioners [11], they increasingly perform case management and other delegated tasks [12]. Tasks in primary care case management that are typically performed by HCAs are regular patient care planning and monitoring, as well as patient education to support self-management [13]. Several randomized controlled trials (RCT) in general practices have indicated that a complex intervention that includes components of primary care case management can improve patient-relevant outcomes compared to usual care, e.g. in patients with major depression [14], with chronic heart failure [15], and at high risk [16]. A systematic review by Panagioti et al. [17] showed that patient self-management support was associated with small but significant improvements in health outcomes and a reduction in health service utilization. However, the costs and cost-effectiveness of case management interventions alongside RCTs often remain unclear [18] although how to manage the burden of chronic disease is a key question for policy-makers. They are actively seeking interventions leading to better health outcomes but the evidence on cost-effectiveness of case management interventions is still scarce, perhaps as a result of methodological challenges [19, 20].

The objective of this analysis was to evaluate the cost-effectiveness of 24 months of primary care case management for patients with a long-term indication for oral anticoagulation therapy in general practices in the federal states of Hesse and Rhineland-Palatinate, Germany. The manuscript adheres to the Consolidated Health Economic Evaluation Reporting Standards (CHEERS) statement/checklist [21].

## Methods

The analysis is part of the cluster-randomized controlled PICANT trial (Primary Care Management for Optimized Antithrombotic Treatment) that was conducted by the Institute of General Practice, Goethe-University Frankfurt am Main, Germany, between June 2012 and March 2015. The aim of the PICANT study was to investigate whether a complex intervention can improve antithrombotic management in primary health care by reducing major thromboembolic and bleeding events compared to usual care. The study protocol reporting the primary and secondary outcomes of the PICANT trial has been published elsewhere [22], as are details of the practice and patient-recruiting process and the results of the screening [23]. In brief, 52 general practices and 736 patients of ≥18 years of age, with a long-term (lifelong) indication for oral anticoagulation, and who were receiving an OAC therapy (e.g. coumarin, dabigatran, rivaroxaban), participated in the PICANT trial. At baseline, 680 (92.4%) had German statutory health insurance (SHI), compared with approximately 90% in the German population as a whole. These patients were considered for the economic analysis because costs were assessed from the perspective of statutory health funds.

### Intervention

The complex intervention in the PICANT trial consisted of a best practice model that included major elements of case management, and patient education tools (e.g. information brochures and a video developed by Hua et al. [24]) for patients with a long-term indication for OAC [25]. We trained HCAs and GPs in case management and regularly monitored patients using the Coagulation-Monitoring-List to improve practice routines [26]. The main elements of the monitoring sessions were to inform patients about their disease and treatment conditions, to encourage patients to perform self-management of oral anticoagulation if they were taking coumarins, and to monitor symptoms and adherence to antithrombotic treatment. HCAs were also trained to detect complications early and to assess adverse events, such as major or minor thromboembolisms or bleeding complications, as well as drug-related side effects and interactions. The HCAs reported the monitoring results to the GP, who decided whether any further action was necessary.

### Data collection and calculation

Cost data was collected using the case report form (CRF), the patient questionnaire, and an additional case management questionnaire for GPs and HCAs for the intervention group only (see Table 1). Data collection started at baseline (T0) and follow-up appraisals were carried out after 12 (T1) and 24 (T2) months. Utility was based on Quality-Adjusted Life Years (QALY) [27] assessed using the generic EuroQol five-dimensional questionnaire (EQ-5D-3 L) [28] included in the patient questionnaire. QALYs were calculated by converting the EQ-5D-3 L health states into utility scores using the German time trade-off scoring algorithm [29]. We used constant price weights to value medical services used and therefore neither cost nor effectiveness outcomes were discounted or adjusted for inflation [30]. Costs and utility were only calculated for non-dropouts with complete data (complete case analysis) [31].

**Table 1:** Cost determinants and unit prices

Resource category	Unit prices in € (year)	Units	Measurement
Direct health care costs			
Outpatient services^a^		Consultations per quarter	Patient questionnaire
*general practitioner*	19.07 (2012), 29.07 (2013), 29.48 (2014/2015)		
*cardiologist*	90.46 (2012/2013), 91.73 (2014/2015)		
*neurologist*	23.30 (2012/2013), 23.63 (2014/2015)		
Outpatient laboratory tests^a^	0.60 (2012–2015)	Consultations per quarter	Case report form
Outpatient prescription of antithrombotic medications^b^		per quarter	Case report form
*phenprocoumon*	16.06 (2012–2015)		
*warfarin*	16.10 (2012–2015)		
*dabigatran*	267.90 (2012–2015)		
*rivaroxaban*	291.79 (2012–2015)		
*apixaban (2.5 mg)*	81.48 (2012–2015)		
*apixaban (5 mg)*	271.00 (2012–2015)		
*acetylsalicylic acid*	0.00 (2012–2015)		
Medical devices^b^		per unit	Case report form/patient questionnaire
*compression therapy*	41.42 (2012–2015)		
*patient self-testing INR*	203.50 (2012–2015)		
Hospital care^c^	depending on diagnosis and length of stay	per stay	Case report form/attachment of anonymized copies of hospital discharge letters including diagnosis and length of stay
Inpatient rehabilitation^d,e^	121.85 (2012–2015)	per day	Case report form/attachment of anonymized copies of discharge letters
Outpatient rehabilitation^d^	46.68 (2012–2015)	per day	Case report form/patient questionnaire
Sick-pay (transfer payments)^f^	depending on insuree’s monthly gross earnings	per day	Patient questionnaire
(Loss of) contributions to statutory social securities^f^	depending on insuree’s monthly gross earnings	per day	Patient questionnaire
Intervention only			
Costs of training and conducting of case management			Case Management questionnaire
*GP*^*g*^	54.62 (2012–2015)	per working hour	
*HCA*^*g*^	15.25 (2012–2015)	per working hour	

^a^Calculations based on the physicians’ fee scale (Uniform Value Scale – EBM) [55, 56]

^b^Prices were taken from Lauer-Taxe® pharmaceutical price information [57]

^c^Calculated on the basis of the official German Diagnoses Related Groups (DRG) reimbursement (2013) [58]

^d^Prices were taken from Bock et al. [59]

^e^According to the German Social Code Book VI, the statutory pension insurance covers rehabilitation services of insured employees. Amongst others, this regulation does not apply to retirees

^f^According to the German Social Code Book V, the SHI covers 70% of the insuree’s monthly gross earnings after 6 weeks of incapacity for work. For the first 6 weeks, the employer continues to pay the salary. During the period of incapacity for work, the health fund also covers 50% of the insuree’s contributions to other statutory insurance programs (pension, long-term care, unemployment and occupational accident). The insuree does not have to pay contributions to SHI during this period. There are several exceptions to these regulations, especially for retirees

^g^Prices were taken from the income survey of the German Federal Statistical Office [60]

### Cost determinants by resource category

To perform the economic analysis from the perspective of statutory health funds, we assessed resource usage using cost determinants recommended by Krauth [32], as shown in Table 1.

Only disease-related costs associated with the patients’ main indication for OAC therapy were evaluated and all costs were calculated in Euros (€). Unit prices were taken from official lists and public sources (see Table 1). All unit prices included rebates and patient co-payments to determine the level of reimbursement relevant for the health funds [33]. For the intervention group, we assessed the resource usage based on the cost determinants applied by Baron et al. [34].

### Statistical analyses

To take into account the skewed distribution of the cost data, 95% confidence intervals (CI) for the mean differences between intervention and control group costs were calculated using the 2.5% and 97.5% percentiles from the non-parametric bootstrapping with 1000 replicates [35]. To adjust for the clustered structure of the data, we drew 26 general practices with replacement per group and calculated unweighted means of costs and QALYs for all patients within those practices in each bootstrap sample. The incremental cost-effectiveness ratio (ICER) was calculated as the ratio of differences in mean total costs and mean number of QALYs between the intervention and the control group [31]. For the bootstrapped data, mean differences between groups were plotted on a cost-effectiveness plane. Furthermore, we calculated the cost-effectiveness acceptability curve (CEAC), which indicates the probability that the intervention was cost-effectiveness at different thresholds of “willingness-to-pay” for an additional QALY [31].

We conducted sensitivity analyses following the example of Hernández et al. [36], who explored the extent to which participants with very high costs influence the cost-effectiveness. We therefore excluded patients with total costs above the 95th and 90th percentile in each study group, respectively, and repeated the analyses. All statistical analyses were performed using IBM SPSS Statistics (version 20 or higher) and R (version 3.4.2).

## Results

*N* = 505 patients (505/680, 74%) were included in the cost analysis because they had SHI, did not drop out of the trial, and could provide cost and QALY data. Their baseline characteristics are presented in Table 2. Participants in the intervention and control groups were comparable in terms of sex, age, indication for oral anticoagulation therapy, type of antithrombotic medication, and EQ-5D score.

**Table 2:** Patient characteristics at baseline (complete case analysis)

Patient characteristics	Intervention (*n* = 258)	Control (*n* = 247)
Mean age (year (SD))	73 (9.7)	72 (9.1)
Sex		
*Male*	142 (55%)	129 (52%)
*Female*	116 (45%)	118 (48%)
Retired	225 (87%)	201 (81%)
Long-term indication for oral anticoagulation therapy		
*atrial fibrillation / flutter*	211 (82%)	187 (76%)
*recurrent venous thromboembolism*	17 (6%)	23 (9%)
*recurrent pulmonary embolism*	3 (1%)	6 (2%)
*mechanical heart prosthesis*	20 (8%)	20 (8%)
*intracardiac thrombus*	2 (1%)	1 (1%)
*other indication*	5 (2%)	10 (4%)
Antithrombotic medication^a^		
*Coumarin*	242 (94%)	232 (94%)
*Dabigatran*	8 (3%)	1 (0.5%)
*Rivaroxaban*	6 (2%)	11 (4%)
*acetylsalicylic acid*	2 (1%)	2 (1%)
*no antithrombotic medication*	0 (0%)	1 (0.5%)
INR self-measuring and dose adjustment (for coumarins only)	27 (11%)	35 (14%)
Patients with migration background	27 (11%)	14 (6%)
EQ-5D score (SD)	0.83 (0.21)	0.80 (0.25)

^a^Apixaban had not been approved at baseline

### Costs and effects

After 24 months, there was no statistically significant difference between the intervention and control, either in terms of mean direct health care costs (mean difference €115, 95% CI [− 201; 406]), or with regard to the various categories of direct health care costs. The mean difference in QALYs between the groups was small and not significant (0.03, 95% CI [− 0.04; 0.11]). The mean difference in total costs was statistically significant (€503, 95% CI [188; 794]) due to the costs of case management that only applied to the intervention group. These results are shown in Table 3.

**Table 3:** Mean (SD) direct health care and intervention costs as well as QALYs per patient after 2 years

Category	Mean cost in € (SD)		Mean difference [95% CI]^a^
	Intervention (*n* = 258)	Control(*n* = 247)	
Physician outpatient care	318 (81)	327 (83)	−9 [−23; 5]
Laboratory tests	5 (1)	5 (1)	−0.1 [− 0.2; 0.1]
Antithrombotic medication	255 (470)	274 (473)	−19 [−105; 62]
Medical devices	42 (108)	38 (96)	4 [−12; 22]
Hospital care	455 (1799)	317 (1317)	138 [− 151; 400]
Sum of direct health care costs	1075 (1974)	960 (1408)	115 [−201; 406]
Case management program (intervention only)	388 (106)	–	–
Sum of total costs	1463 (1979)	960 (1408)	503 [188; 794]
QALY^b^	1.63 (0.40)	1.59 (0.43)	0.03 [− 0.04; 0.11]

^a^ Calculated from bootstrapped dataset

^b^ Calculation based on the EQ-5D^Index^ for Germany [29]

Cost drivers in both groups were costs for hospital care (≥ 40%), for physician outpatient care (≥ 25%), and for oral anticoagulation medication (≥ 23%). The intervention costs per patient were approximately €388 after 24 months, comprising higher costs in the first year (€215) and lower costs in the second year (€175). Although relevant for statutory health insurers, costs for rehabilitation services (outpatient and inpatient), sick pay (transfer payments) for employees, and loss of patients’ contributions to SHI and other statutory insurance programs were not assessed in the economic analysis because the amounts concerned were negligible in this study population, ≥ 81% of whom were retirees.

### Cost-effectiveness

**Fig. 1:**
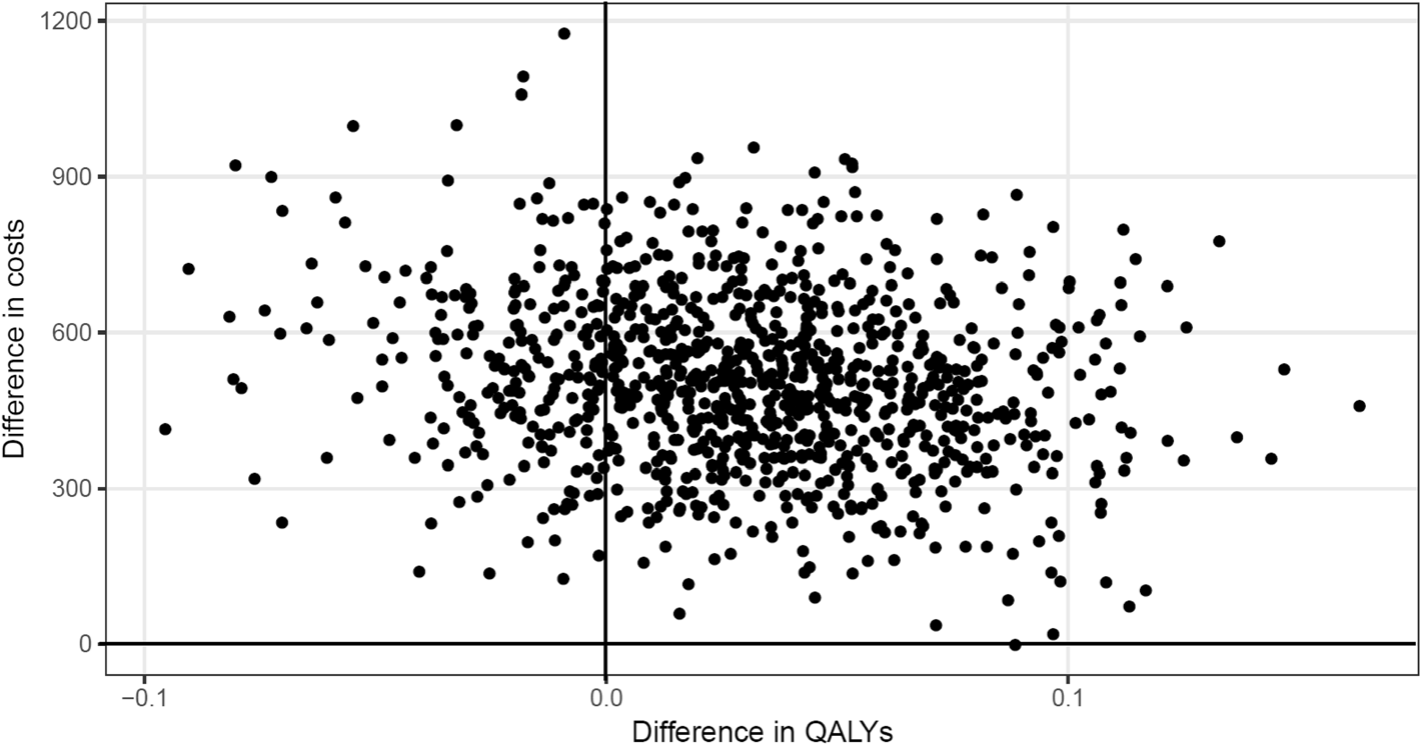
Distribution of bootstrapped incremental total costs and QALYs

The ICER was €16,767 per QALY. Figure 1 presents the bootstrapped results in the intervention and control groups displayed in a cost-effectiveness plane.

**Fig. 2:**
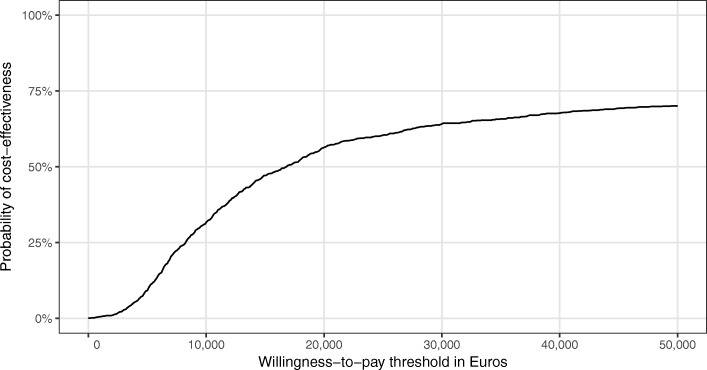
Cost-effectiveness acceptability curve (CEAC)

It shows that the intervention was more effective regarding QALYs than usual care, but was also more costly. Of the bootstrapped ICERs, the majority (more than 75%) indicated an increase in QALYs at an incremental cost, whereas only around 25% indicated a decrease in QALYs at an incremental cost. The resulting CEAC (see Fig. 2) shows that the probability of the intervention being cost-effective never rose above 70%, regardless of health insurer’s willingness to pay per QALY. If the health insurer was willing to pay €15,000 per additional QALY, the probability of cost-effectiveness was 50%.

### Sensitivity analyses

The results of the sensitivity analyses are presented in Table 4.

**Table 4:** Results of the sensitivity analyses

Category	5% excluded	10% excluded
	Mean difference ^a^ [95% CI]^b^	Mean difference ^a^ [95% CI]^b^
Direct health care costs (€)	4 [− 120; 100]	−15 [− 90; 59]
Total costs (€)	392 [267; 485]	372 [299; 445]
QALY	0.03 [−0.04; 0.11]	0.04 [−0.04; 0.11]
ICER	13,067	9300

^a^ Mean differences are intervention - control group

^b^ Calculated from bootstrapped dataset

When the 5% of participants that generated the highest costs were excluded, no statistically significant differences existed between the groups in terms of either direct health care costs, or QALYs. The results remained similar when the 10% of participants that were responsible for the highest cost were excluded. In terms of total costs, the results were only statistically significant because of the additional case management costs relating to the intervention group. However, the sensitivity analyses had only minimal effects on the incremental cost-effectiveness ratio.

## Discussion

In this analysis, we compared cost-effectiveness after 24 months of primary care case management in German general practices for patients with a lifelong indication for OAC therapy with usual care. The mean difference in direct health care costs and QALYs between the two groups was small and not significant. The difference in mean total costs per patient was statistically significant as the costs of case management were only relevant in the intervention group. The ICER was €16,767 per QALY, and the probability of the intervention being cost-effective never rose above 70%, regardless of payer willingness to pay for each QALY.

Several studies have indicated that case management interventions can improve patient-relevant outcomes [7, 9]. This holds also true for the PICANT trial. As a secondary objective, we investigated whether the complex intervention leads to an increase in patient knowledge about anticoagulation therapy compared to usual care [22]. After 12 months, the improvement in patients’ knowledge (compared to baseline) was significantly greater in the intervention than in the control group, and the difference between both groups remained statistically significant after 24 months [37]. However, little is known about the economic effects of such complex interventions tested alongside studies with an adequate study design like RCTs [18]. In the SPRING trial, Tiessen et al. [38] assessed the costs and cost-effectiveness of cardiovascular prevention when conducted in patients with an elevated cardiovascular risk by practice nurses in general practice. The results are similar to those of the PICANT trial, as the total costs were higher in the intervention group (mean difference €175 [SPRING trial] vs. €503 [PICANT trial]), and 65% vs. 75% of the bootstrapped ICERs were located in the northeast quadrant. Regardless of a decision maker’s willingness to pay, the probability that the SPRING intervention would be cost effective compared to usual care never rose above 60% (vs. 70% in the PICANT trial). A cost-effectiveness analysis of a HCA-based case management for patients with depression showed no significant differences in QALYs and total costs between intervention and control groups after 24 months [39]. Oksman et al. [40] performed a cost-effectiveness analysis of a tele-based health-coaching program for patients with chronic diseases (type 2 diabetes, coronary artery diseases, and congestive heart failure). Similar to the results of the PICANT trial, the intervention was more effective regarding QALYs than usual care but also more costly. Compared to a HCA-based case management for high-risk patients [16], the cost of training and performing case management in the intervention group was slightly higher in the PICANT trial (€388 vs. US$247, or €211.80 based on the exchange of €1 = US$1.16622 rate on November 13, 2017). However, in both RCTs the costs of case management decreased in the second year because training costs were only relevant at the beginning of the intervention. Kaier et al. [41] performed a budget impact analysis of a telemedically supported case management (intervention) for patients with donor kidney transplantation and the intervention group showed a lower utilization of medical services as well as better medical outcomes. Other economic assessments failed to show that a nurse-based case management was either effective or cost-effective compared to usual care, e.g. for patients recently discharged from intensive care units [36], and for elderly patients with myocardial infarction after 1 year [42]. However, the latter results was revised after 3 years [43].

Several methodological challenges must be confronted when conducting economic evaluations in parallel to RCTs:

- the study duration may be too short to capture relevant economic outcomes [19];
- resources can be consumed for trial purposes only and therefore costs can be overestimated (“protocol driven care”) [19];
- the limited follow-up may alter estimated clinical effectiveness [44];
- when calculated to detect differences in clinical outcomes, the sample size may be too small (“underpowered”) to detect differences in economic indicators [45];
- the generalizability of cost-effectiveness analysis can be threated “[…] when the comparison therapy is not the most relevant for the policy question being addressed.” [31, p., 248];
- the additional cost data collection in RCTs can increase both the costs of clinical trials and the burden on study participants [30].

Based on an analysis of registry data, Reinhold et al. [46] calculated that the direct health care costs covered by SHI of patients with atrial fibrillation in Germany amounted to at least €3274 annually. Although we chose the same perspective, the direct health care costs in the PICANT trial were much lower, possibly, because we only took the costs of oral anticoagulation and not antiplatelet therapies into account. Similar to the PICANT trial, direct health care costs were mainly driven by hospital care [46]. Other studies from Finland [47], USA [48], and Canada [49], reported direct health care costs for patients with atrial fibrillation of between €500 and €600 annually (at the current Euro exchange rates). Nevertheless, these studies only included patients who were taking warfarin. In the PICANT trial, we also included patients who were taking DOACs such as dabigatran, rivaroxaban or apixaban, which are more costly. In Germany, the mean net cost of coumarins is €0.18 per daily defined dose (DDD), compared to €3.75 for dabigatran, and €3.45 for factor Xa antagonists (e.g. rivaroxaban, apixaban) [50]. A recently published health technology assessment from UK [51] aimed to identify the most effective, safe and cost-effective anticoagulant for stroke prevention in patients with atrial fibrillation, and for primary prevention, treatment and secondary prevention of venous thromboembolisms. The results suggested that DOACs have efficacy and safety advantages over warfarin in patients with atrial fibrillation, but no more efficacious when used to treat acute venous thromboembolisms [51]. Of the available DOACs, apixaban had the highest probability of being cost-effective compared to warfarin, with a willingness-to-pay threshold of > £5000 (which corresponds to €5575.50 based on the exchange rate of €1 = £0.8970 on November 15, 2017) [51].

### Strengths and limitations

Although economic evaluations are mostly performed from a societal perspective, this analysis chose the perspective of statutory health insurers. As these sickness funds cover most of the cost of health care in Germany, the results may help health care professionals decide how best to allocate resources, especially for chronically ill patients. Unrelated health care costs did not bias the results of our economic analysis as only disease related health care costs were included. One limitation of our analysis is that utilization and costs are more likely to have been underestimated than overestimated because we used unit prices from official lists and public sources. Furthermore, costs were partly calculated based on patient’s self-reported data on service use. A recall bias may therefore have led to an underestimation of costs. Although the study included a 24-month follow-up, we never used conservative methods to deal with missing data (e.g., data imputation using the last observation carried forward method). Instead of this, we used a complete case analysis. This is a more naive and simple approach to deal with missing data. However, when complete case analyses are used, (mean) cost estimates are always less precise than would be desirable. No adjustment besides the sampling strategy for the bootstrap was made to take the effects of clustering into account. With respect to the calculation of QALYs, alternative utility measurements could also have been considered, such as the 36-item short-form health survey (SF-36) [52] or the Health Utilities Index Mark 2 (HUI2) [53] or Mark 3 (HUI3) [54] but no German-specific utility weights for these measurements yet exist. However, our study is one of very few cost-effectiveness analyses of primary care case management programs for chronically ill patients that have been carried out in a real-life primary care setting as part of an RCT.

## Conclusions

The PICANT trial indicated that primary care case management for patients with a long-term indication for oral anticoagulation therapy improved QALYs compared to usual care, but was also more costly. However, case management did not result in a statistically significant improvement in QALYs or direct health care costs compared to usual care over a period of 24 months. This RCT was conducted under real-life conditions in primary care and may help professionals and policy-makers allocate scarce health care resources in such a way that the overall quality of care is improved at moderate costs, particularly for chronically ill patients, such as those with a long-term indication for OAC therapy. Our study could help to inform the policy debate about whether an effective therapy provides sufficient value for its cost to be adopted for use and to facilitate judgments about health care interventions.

## Abbreviations

CEAC: Cost-effectiveness acceptability curve
CI: Confidence interval
CRF: Case report form
DDD: Daily defined dose
DOAC: Direct oral anticoagulant
DRG: Diagnosis related groups
EQ-5D: EuroQol five-dimensional questionnaire
GP: General practitioner
HCA: Health care assistant
HUI: Health Utilities Index Mark
ICER: Incremental cost-effectiveness ratio
OAC: Oral anticoagulant
PICANT: Primary Care Management for Optimized Antithrombotic Treatment
QALY: Quality adjusted life year
RCT: Randomized controlled trial
SHI: Statutory health insurance
